# Validation List no. 229: valid publication of new names and new combinations effectively published outside the IJSEM

**DOI:** 10.1099/ijsem.0.007141

**Published:** 2026-05-29

**Authors:** Aharon Oren, Markus Göker

**Affiliations:** 1 The Institute of Life Sciences, The Hebrew University of Jerusalem, The Edmond J. Safra Campus, 9190401 Jerusalem, Israel; 2 Leibniz Institute DSMZ – German Collection of Microorganisms and Cell Cultures, Inhoffenstrasse 7B, 38124 Braunschweig, Germany

The purpose of this list is to publish in the International Journal of Systematic and Evolutionary Microbiology (IJSEM) those new names and new combinations that were effectively published outside the IJSEM as set forth in Rule 27 of the International Code of Nomenclature of Prokaryotes (2025 Revision) [1]. Authors and other individuals wishing to have new names and/or combinations included in future lists should send an electronic copy of the published paper to the IJSEM Editorial Office for confirmation that all of the other requirements for valid publication have been met. It is also a requirement of IJSEM and the International Code of Nomenclature of Prokaryotes (ICNP) that authors of new species, new subspecies and new combinations provide evidence that types are deposited in two publicly accessible culture collections in two different countries. It should be noted that the date of valid publication of these new names and combinations is the date of publication of this list, not the date of the original publication of the names and combinations. The authors of the new names and combinations are as given below.

Inclusion of a name on these lists validates the publication of the name and thereby makes it available in the nomenclature of prokaryotes. Inclusion of a name on these lists is part of the requirements of a valid publication of an effectively published name. Indeed, some of these names may, in time, be considered later synonyms, or it may be proposed to transfer the organisms to another genus, thus necessitating the creation of a new combination. Conversely, it may be proposed to transfer an organism back to an earlier genus and to regard the basonym or an earlier new combination as the correct name instead of a more recent new combination. The ICNP does not decide on such taxonomic opinions.

**Table:** 

Name/author	Proposed as	Nomenclatural type^1^	Priority^2^	Reference
*Acetobacter zythi* Bongaerts *et al*. 2026, 13^3,4^	sp. nov.	LMG 32666 (=CECT 30722)	18	[2]
*Acinetobacter mesopotamicus* Acer *et al*. 2020, 3198	sp. nov.	GC2 (=DSM 26953=JCM 31073)	93	[3]
*Acinetobacter pullorum* Elnar *et al*. 2020, 531	sp. nov.	B301 (=JCM 33942=KACC 21653)	98	[4]
*Actinomyces oralis* Mbogning Fonkou *et al*. 2018, 43^5^	sp. nov.	Marseille-P3109 (=CSUR P3109=DSM 103942)	75	[5]
*Adhaeribacter soli* Han *et al*. 2021, 167	sp. nov.	MA2 (=KCTC 72630=NBRC 114192)	80	[6]
*Agrobacterium vacciniicorymbosi* Iličić *et al*. 2025, 10^3^	sp. nov.	BA1120 (=CFBP 9290=NCPPB 4800)	129	[7]
*Algorimicrobium* García-López *et al*. 2019, 29^3^	gen. nov.	*Algorimicrobium algoritergicola*	135	[8]
*Algorimicrobium algoritergicola* (Bowman and Nichols 2005) García-López *et al*. 2019, 30^3^	comb. nov. (basonym: *Bizionia algoritergicola* Bowman and Nichols 2005)	APA-1 (=CIP 108533=KMM 8430)^6^	135	[8]
*Algorimicrobium bowmanii* Kurilenko *et al*. 2026, 16^3^	sp. nov.	041–53-Ur-6 (=KCTC 72011=KMM 8389)	136	[9]
*Algorimicrobium echini* (Nedashkovskaya *et al*. 2010) García-López *et al*. 2019, 30^3^	comb. nov. (basonym: *Bizionia echini* Nedashkovskaya *et al*. 2010)	KMM 6177 (=DSM 23925=KCTC 22015)	135	[8]
*Alistipes intestinihominis* Hitch *et al*. 2025, 11^3^	sp. nov.	CLA-KB-H122 (=DSM 118481=JCM 38255)	123	[10]
*Alistipes provencensis* Bellali *et al*. 2019, 8^3,7^	sp. nov.	Marseille-P2431 (=CSUR P2431=DSM 102308)	55	[11]
*Antarctobacter albus* corrig. (Zheng *et al*. 2010) Xu *et al*. 2025, 11^3,8^	comb. nov. (basonym: *Mameliella alba* Zheng *et al*. 2010)	JLT354-W (=CGMCC 1.7290=LMG 24665)	1	[12]
*Antarctobacter aquimaris* (Jung *et al*. 2016) Xu *et al*. 2025, 11^3^	comb. nov. (basonym: *Maliponia aquimaris* Jung *et al*. 2016)	MM-10 (=CECT 8898=KCTC 42721)	1	[12]
*Antarctobacter sediminis* (Zheng *et al*. 2022) Xu *et al*. 2025, 11^3^	comb. nov. (basonym: *Mameliella sediminis* Zheng *et al*. 2022)	DP3N28-2 (=KCTC 82804=MCCC 1K06218)	1	[12]
*Aquibium pacificum* corrig. Jiang *et al*. 2024, 10^3,9^	sp. nov.	LZ166 (=KCTC 82889=MCCC M28807)^10^	14	[13]
*Bacillus bingmayongensis* Liu *et al*. 2014, 507^11^	sp. nov.	FJAT-13831 (=CGMCC 1.12043=DSM 25427)	65	[14]
*Bacillus cheonanensis* Kim *et al*. 2014, 557	sp. nov.	PFS-5 (=JCM 19333=KACC 17469)	39	[15]
*Bacillus dabaoshanensis* Cui *et al*. 2015, 519^12^	sp. nov.	GSS04 (=CCTCC AB 2013260=KCTC 33191)	90	[16]
*Bacteroides ihuae* Andrieu *et al*. 2018, 86	sp. nov.	Marseille-P2824 (=CCUG 70550=CSUR P2824)	70	[17]
*Brevibacillus antibioticus* Choi *et al*. 2019, 995	sp. nov.	TGS2-1 (=FBCC-B2501=NBRC 113840)^13^	59	[18]
*Bruguierivorax* Li *et al*. 2021, 862^14^	gen. nov.	*Bruguierivorax albus*	115	[19]
*Bruguierivorax albus* Li *et al*. 2021, 862	sp. nov.	BGMRC 2031 (=KCTC 52119=NBRC 111907)	115	[19]
*Burkholderia alba* Lee *et al*. 2018, 315	sp. nov.	AD18 (=JCM 32403=KCCM 43268)	108	[20]
*Campylobacter felis* Wang *et al*. 2023, 12^3^	sp. nov.	XJK22-1 (=GDMCC 1.3684=JCM 35847)	133	[21]
*Campylobacter ovis* Wang *et al*. 2023, 12^3^	sp. nov.	SYS25-1 (=GDMCC 1.3685=JCM 35848)	52	[21]
*Cellulomonas triticagri* Han *et al*. 2022, 6^3^	sp. nov.	NEAU-YY56 (=DSM 106717=JCM 32550)	15	[22]
*Chelatococcus thermostellatus* Ibrahim *et al*. 2016, 8^3,15^	sp. nov.	MW9 (=DSM 28244=LMG 27009)	145	[23]
*Chitinophaga soli* An *et al*. 2007, 708	sp. nov.	Gsoil 219 (=KCTC 12650=LMG 23593)^16^	68	[24]
*Chryseobacterium formosum* corrig. Akter *et al*. 2015, 1015^17^	sp. nov.	THG-DN3.6 (=CCTCC AB 2015118=KCTC 42606)^18^	51	[25]
*Chryseobacterium panacis* Singh *et al*. 2016, 193	sp. nov.	DCY107 (=CCTCC AB 2015195=KCTC 42750)	60	[26]
*Citrobacter arsenatis* Wang *et al*. 2021, 1291^19^	sp. nov.	LY-1 (=CCTCC AB 2019169=KCTC 72440)	120	[27]
*Citrobacter tructae* Jung *et al*. 2021, 16^3^	sp. nov.	SNU WT2 (=JCM 33612=KCTC 72517)^20^	6	[28]
*Clostridium tunisiense* Thabet *et al*. 2004, 189^21^	sp. nov.	E1 (=CIP 107666=DSM 15206)	36	[29]
*Coraliitalea* Yoon *et al*. 2018, 469	gen. nov.	*Coraliitalea coralii*	73	[30]
*Coraliitalea coralii* Yoon *et al*. 2018, 469	sp. nov.	04OKA-3–121 (=KCTC 52378=NBRC 112329)	73	[30]
*Corynebacterium hiratae* de Oliveira Sant’Anna *et al*. 2024, 1412	sp. nov.	332 (=CCBH 35014=CCCT 24.09)^22^	26	[31]
*Desulfobulbus aggregans* Kharrat *et al*. 2017, 454	sp. nov.	SM40 (=DSM 28693=JCM 19994)	134	[32]
*Desulfomicrobium aggregans* Dengler *et al*. 2023, 8^3^	sp. nov.	CaP3V-S-L1A (=DSM 113299=JCM 39179)	28	[33]
*Desulfosporosinus cupriresistens* Karnachuk *et al*. 2025, 8^3^	sp. nov.	BG (=JCM 32882=VKM B-3258)	103	[34]
*Desulfovibrio brasiliensis* Warthmann *et al*. 2005, 260^23^	sp. nov.	LVform1 (=DSM 15816=JCM 12178)^24^	46	[35]
*Dyella thailandensis* Intarapasit *et al*. 2026, 9^3^	sp. nov.	KULCS107 (=KCTC 18276=NBRC 117363=TBRC 20486)^25^	128	[36]
*Dysgonomonas massiliensis* Bilen *et al*. 2019, 943	sp. nov.	Marseille-P4356 (=CCUG 71356=CSUR P4356)	111	[37]
*Faecalibacterium tardum* Hitch *et al*. 2025, 13^3^	sp. nov.	CLA-AA-H175 (=DSM 116192=JCM 39670)	123	[10]
*Flavisolibacter aluminii* Lee *et al*. 2019, 21	sp. nov.	ID1709 (=KCTC 52778=NBRC 112870)^26^	22	[38]
*Flavisolibacter galbus* Maeng *et al*. 2019, 1564	sp. nov.	17J28-26 (=JCM 33203=KCTC 62222)	23	[39]
*Flavobacterium agricola* Lin *et al*. 2023, 1355^27^	sp. nov.	CC-SYL302 (=BCRC 81320=JCM 34764)	118	[40]
*Flavobacterium panacis* Kim *et al*. 2016, 1205	sp. nov.	DCY106 (=JCM 31468=KCTC 42747)	9	[41]
*Foetidibacter* Pu *et al*. 2021, 2429	gen. nov.	*Foetidibacter luteolus*	76	[42]
*Foetidibacter luteolus* Pu *et al*. 2021, 2429	sp. nov.	YG09 (=KCTC 72595=MCCC 1K04042)	76	[42]
Frigoribacterium adelgis Tamošiūnaitė *et al*. 2026, 12^3,28^	sp. nov.	D8 (=DSM 119719=LMG 34012)	127	[43]
*Fusobacterium hwasookii* Cho *et al*. 2015, 174	sp. nov.	ChDC F128 (=JCM 30218=KCTC 5108)^29^	30	[44]
*Fusobacterium pseudoperiodonticum* Park *et al*. 2019, 663^30^	sp. nov.	ChDC F213 (=JCM 33009=KCTC 5677)^31^	13	[45]
*Gluconacetobacter brunescens* Karničnik *et al*. 2025, 14^3,32^	sp. nov.	Hr-1–5 (=LMG 33629=ZIM B1168)	2	[46]
*Halobacillus rhizosphaerae* Yin *et al*. 2025, 8^3^	sp. nov.	T66 (=JCM 36534=MCCC 1K08701)	89	[47]
*Halomonas ethanolica* Wang and Shao 2021, 14^3^	sp. nov.	CYT3-1-1 (=KCTC 72090=MCCC 1A11081)	81	[48]
*Halomonas zhangzhouensis* Wang and Shao 2021, 13^3^	sp. nov.	CXT3-11 (=KCTC 72087=MCCC 1A11036	81	[48]
*Halonovum* Hwang *et al*. 2026, 10^3^	gen. nov.	*Halonovum salinarum*	125	[49]
*Halonovum salinarum* Hwang *et al*. 2026, 10^3^	sp. nov.	MBLA0143 (=JCM 36640=KCTC 4317)	125	[49]
*Halonovum rutilum* Hwang *et al*. 2026, 10^3^	sp. nov.	MBLA0147 (=JCM 36641=KCTC 4319)	125	[49]
*Herbivorax alkaliphila* Sorokin *et al*. 2026, 11^3^	sp. nov.	ANBcel31 (=DSM 113934=UQM 41585)	114	[50]
*Hoeflea siderophila* Sorokina *et al*. 2012, 64^33^	sp. nov.	Hf1 (=DSM 21587=UQM 40581)^34^	47	[51]
*Hominisplanchenecus murintestinalis* Afrizal *et al*. 2022, 16^3^	sp. nov.	CLA-AA-M05 (=DSM 111139=JCM 38251)	104	[52]
*Hydrogenophaga temperata* Kim *et al*. 2010, 423^35^	sp. nov.	TR7-01 (=DSM 18117=KCTC 12203)	97	[53]
*Hymenobacter busanensis* Lee *et al*. 2021, 760^36^	sp. nov.	MA3 (=KCTC 72631=NBRC 114193)	78	[54]
*Janthinobacterium psychrotolerans* Gong *et al*. 2017, 5^3^	sp. nov.	S3-2 (=DSM 102223=LMG 29653)	144	[55]
*Jeotgalibacillus aurantiacus* Jiang *et al*. 2022, 780	sp. nov.	T12=KCTC 43296=MCCC 1K07171)	82	[56]
*Kluyvera chengduensis* Liu *et al*. 2026, 6^3,37^	sp. nov.	142359 (=GDMCC 1.5094=JCM 37737)	146	[57]
*Kluyvera huaxiensis* Liu *et al*. 2026, 4^3,37^	sp. nov.	142053 (=GDMCC 1.4845=JCM 37413	146	[57]
*Lachnospira intestinalis* Hitch *et al*. 2025, 13^3^	sp. nov.	CLA-AA-H89B (=DSM 118070=JCM 38254)	123	[10]
*Lacisediminihabitans changchengi* Liang *et al*. 2021, 5523	sp. nov.	G11-30 (=CCTCC AA 2019080=KCTC 49359)	77	[58]
*Lautropia dentalis* Lim *et al*. 2019, 1371	sp. nov.	ChDC F240 (=JCM 33297=KCOM 2505)	99	[59]
*Litorerythrobacter* Kathiresan *et al*. 2026, 10^3^	gen. nov.	*Litorerythrobacter xanthomarinus*	101	[60]
*Litorerythrobacter xanthomarinus* Kathiresan *et al*. 2026, 10^3^	sp. nov.	MF3-039 (=KCTC 8705=KEMB 23948=TBRC 19385)	101	[60]
*Lotipaludicoccus* Park *et al*. 2026, 7^3^	gen. nov.	*Lotipaludicoccus stagni*	139	[61]
*Lotipaludicoccus stagni* Park *et al*. 2026, 7^3^	sp. nov.	IMCC34717 (=KACC 23134=KCTC 82067=NBRC 113911)	139	[61]
*Luteimonas granuli* Siddiqi *et al*. 2020, 2006	sp. nov.	Gr-4 (=JCM 18203=KACC 16614)	21	[62]
*Lysobacter arenosi* Kim *et al*. 2021, 715	sp. nov.	R7 (=JCM 34257=KACC 21663)	20	[63]
*Lysobacter chinensis* Liu *et al*. 2022, 1038	sp. nov.	TLK-CK17 (=CCTCC AB 2021257=KCTC 92122)	48	[64]
*Lysobacter pedocola* Jang *et al*. 2018, 391	sp. nov.	IPC6 (=JCM 31020=KCTC 42811)	12	[65]
*Lysobacter solisilvae* Kim *et al*. 2021, 715^38^	sp. nov.	R19 (=JCM 34258=KACC 21767)	20	[63]
*Mangrovimonas xylaniphaga* Dinesh *et al*. 2017, 66	sp. nov.	ST2L12 (=JCM 30880=LMG 28914)	10	[66]
*Maridesulfovibrio gilichinskyi* (Ryzhmanova *et al*. 2019) Galushko and Kuever 2021, 7^3^	comb. nov. (basonym: *Desulfovibrio gilichinskyi* Ryzhmanova *et al*. 2019)	K3S (=DSM 100341=VKM B-2877)	58	[67]
*Marinicella algicola* Bayburt *et al*. 2026, 11^3^	sp. nov.	W31 (=JCM 35965=KACC 23129)	8	[68]
*Marinimicrobium hydrolyticum* Sorokin *et al*. 2025, 9^3^	sp. nov.	ABcell2 (=DSM 115774=JCM 35976)	112	[69]
*Marinitalea* Kim *et al*. 2011, 92	gen. nov.	*Marinitalea sucinacia*	45	[70]
*Marinitalea sucinacia* Kim *et al*. 2011, 92^39^	sp. nov.	JC2131 (=JCM 14003=KCTC 12705)	45	[70]
*Marinovum sedimenti* Romanenko *et al*. 2026, 14^3^	sp. nov.	KMM 9989 (=KCTC 8835)	132	[71]
*Marisediminitalea alba* (Sun *et al*. 2019) Bayburt *et al*. 2026, 12^3^	comb. nov. (basonym: *Alteromonas alba* Sun *et al*. 2019)	190 (=KCTC 62227=MCCC 1K03456)	8	[68]
*Marisediminitalea lipolytica* (Shi *et al*. 2017) Bayburt *et al*. 2026, 12^3^	comb. nov. (basonym: *Alteromonas lipolytica* Shi *et al*. 2017)	JW12 (=CGMCC 1.15735=KCTC 52408)	8	[68]
*Marisediminitalea oceani* (Jin *et al*. 2018) Bayburt *et al*. 2026, 12^3^	comb. nov. (basonym: *Alteromonas oceani* Jin *et al*. 2018)	S35 (=CGMCC 1.16029=KCTC 52449)	8	[68]
*Mesobacterium hydrothermale* Su *et al*. 2024, 7^3^	sp. nov.	TK19101 (=KCTC 8354=MCCC 1K08936)	87	[72]
*Mesorhizobium koreense* Lee *et al*. 2024, 1822	sp. nov.	WR6 (=KCTC 92695=NBRC 116021)	16	[73]
*Mesorhizobium maamorense* Alami *et al*. 2026, 12^3^	sp. nov.	ORM16 (=CCMM B1359=DSM 120599)	122	[74]
*Mesosutterella faecium* Yu *et al*. 2024, 13^3.40^	sp. nov.	AGMB02718 (=GDMCC 1.2717=KCTC 25541)^41^	67	[75]
*Metabacillus elymi* Lee *et al*. 2021, 1716	sp. nov.	KUD1714 (=DSM 27608=KCTC 33222)	24	[76]
*Methanofollis propanolicus* Dengler *et al*. 2022, 4^3,42^	sp. nov.	CaP3V-MF-L2A (=DSM 113321=JCM 39176)	7	[77]
*Methylobacter alkaliphilus* corrig. Khmelenina *et al*. 1997, 260^43^	sp. nov.	20Z (=DSM 19304=VKM B-2133)	94	[78]
*Methylobacterium flocculans* Gao *et al*. 2024, 7^3^	sp. nov.	FF17 (=KCTC 8320=MCCC 1K08738)	83	[79]
*Micromonospora jinlongensis* Gao *et al*. 2014, 311^44^	sp. nov.	NEAU-GRX11 (=CGMCC 4.7103=DSM 45876)	69	[80]
*Microvirga pudoricolor* Oh *et al*. 2021, 6075	sp. nov.	BT291 (=KCTC 72368=NBRC 114845)	79	[81]
*Mucilaginibacter hankyongensis* Liu *et al*. 2017, 528	sp. nov.	BR5-28 (=DSM 21151=KCTC 22274)	5	[82]
*Muricauda lutisoli* Jeong *et al*. 2022, 5^3^	sp. nov.	CAU 1631 (=KCTC 82456=MCCC 1K06088)	85	[83]
*Natrarchaeobius oligotrophus* Tulenkov *et al*. 2025, 10^3^	sp. nov.	Aarcht7 (=DSM 119936=UNIQEM U967)^45^	113	[84]
*Natrarchaeobius versutus* Tulenkov *et al*. 2025, 10^3,46^	sp. nov.	AArcel7 (=DSM 119677=UNIQEM U973)^47^	113	[84]
*Natronocytophaga* Sorokin *et al*. 2025, 9^3^	gen. nov.	*Natronocytophaga cellulosiphila*	112	[69]
*Natronocytophaga cellulosiphila* Sorokin *et al*. 2025, 9^3^	sp. nov.	ABcell3 (=DSM 115919=UQM 41578)	112	[69]
*Neoalteromonas* Bayburt *et al*. 2026, 13^3,48^	gen. nov.	*Neoalteromonas facilis*	8	[68]
*Neoalteromonas arenosi* (Luo *et al*. 2025) Bayburt *et al*. 2026, 13^3,49^	comb. nov. (basonym: *Alteromonas arenosi* Luo *et al*. 2025)^49^	ASW11-36 (=KCTC 82496=MCCC 1K05585)	8	[68]
*Neoalteromonas facilis* (Zhang *et al*. 2020) Bayburt *et al*. 2026, 13^3^	comb. nov. (basonym: *Alteromonas facilis* Zhang *et al*. 2020)	P0213 (=KCTC 62079=MCCC 1H00243)	8	[68]
*Neoalteromonas flava* (Zhang *et al*. 2020) Bayburt *et al*. 2026, 13^3^	comb. nov. (basonym: *Alteromonas flava* Zhang *et al*. 2020)	P0211 (=KCTC 62078=MCCC 1H00242)	8	[68]
*Niallia hominis* Hitch *et al*. 2025, 15^3^	sp. nov.	CLA-SR-H024 (=DSM 118483=JCM 38256)	123	[10]
*Ningiella algicola* Bayburt *et al*. 2026, 11^3^	sp. nov.	W23 (=JCM 35964=KACC 23126)	8	[68]
*Nocardioides jejuensis* Jang *et al*. 2020, 344	sp. nov.	18JY15-6 (=JCM 33182=KCTC 49105)	11	[85]
*Nocardioides paucivorans* Ahn *et al*. 2014, 993	sp. nov.	KIS31-44 (=DSM 27142=KACC 17309)	37	[86]
*Nocardioides suum* Lee *et al*. 2017, 420	sp. nov.	PBT33-2 (=DSM 102833=KCTC 39558)	31	[87]
*Nocardiopsis oceanisediminis* Kim *et al*. 2025, 6^3^	sp. nov.	CNT-189 (=KACC 22136=NBRC 114760)	138	[88]
*Novosphingobium tardum* Chen *et al*. 2015, 55	sp. nov.	16-28-2 (=CGMCC 1.12989=NBRC 110956)	32	[89]
*Oceanisphaera pacifica* Xue *et al*. 2022, 7^3,50^	sp. nov.	DM8 (=KCTC 82764=MCCC 1K06133)	84	[90]
*Oceanobacillus gochujangensis* Jang *et al*. 2014, 1054^51^	sp. nov.	TK1655 (=CIP 110582=KCTC 33014=NBRC 109637)^52^	42	[91]
*Oceanobacillus jeddahensis* corrig. Khelaifia *et al*. 2016, 256^53^	sp. nov.	S5 (=CSUR P1091=DSM 28586)	54	[92]
*Oceanobacillus massiliensis* Roux *et al*. 2013, 381	sp. nov.	N’diop (=CSUR P132=DSM 24644)^54^	74	[93]
*Orenia metallireducens* Dong *et al*. 2016, 6451^55^	sp. nov.	Z6 (=ATCC BAA-2645=JCM 31419)	119	[94]
*Paenibacillus bouchesdurhonensis* Pham *et al*. 2017, 10^56^	sp. nov.	Marseille-P3071 (=CSUR P3071=DSM 103972)	71	[95]
*Paenibacillus lycopersici* Lee *et al*. 2020, 838	sp. nov.	12200R-189 (=CCTCC AB 2020027=KACC 19916)	57	[96]
*Paenibacillus phocaeensis* corrig. Tidjani Alou *et al*. 2017, 21^57^	sp. nov.	mt24 (=CSUR P2238=DSM 101777)	44	[97]
*Paenibacillus tuaregi* Pham *et al*. 2017, 12^58^	sp. nov.	Marseille-P2472 (=CSUR P2472=DSM 102801)	71	[95]
*Paludibacterium flexuosum* Nakamura *et al*. 2026, 11^3^	sp. nov.	THUN1379 (=JCM 36560=KCTC 8465)	130	[98]
*Paracoccus zhejiangensis* Wu *et al*. 2013, 125	sp. nov.	J6 (=CCTCC AB 2012031=KACC 16703)	61	[99]
*Paralteromonas* Bayburt *et al*. 2026, 13^3^	gen. nov.	*Paralteromonas sediminis*	8	[68]
*Paralteromonas sediminis* (Ye *et al*. 2019) Bayburt *et al*. 2026, 13^3^	comb. nov. (basonym: *Alteromonas sediminis* Ye *et al*. 2019)	U0105 (=KCTC 62080=MCCC 1H00299)	8	[68]
*Paraniabella* Kim *et al*. 2025, 9^3^	gen. nov.	*Paraniabella ginsengisoli*	105	[100]
*Paraniabella aurantiaca* Kim *et al*. 2025, 10^3^	sp. nov.	CJ426 (=JCM 37728=KACC 23908)	105	[100]
*Paraniabella defluvii* (Min *et al*. 2024) Kim *et al*. 2025, 11^3^	comb. nov. (basonym: *Niabella defluvii* Min *et al*. 2024)	I65 (=JCM 35315=KACC 22647)	105	[100]
*Paraniabella ginsengisoli* (Weon *et al*. 2009) Kim *et al*. 2025, 11^3^	comb. nov. (basonym: *Niabella ginsengisoli* Weon *et al*. 2009)	GR10-1 (=JCM 15444=KACC 13021)	105	[100]
*Paraniabella hibiscisoli* (Ngo *et al*. 2017) Kim *et al*. 2025, 11^3^	comb. nov. (basonym: *Niabella hibiscisoli* Ngo *et al*. 2017)	THG-DN5.5 (=CCTCC AB 2016086=KACC 18857)	105	[100]
*Paraniabella yanshanensis* (Wang *et al*. 2009) Kim *et al*. 2025, 11^3^	comb. nov. (basonym: *Niabella yanshanensis* Wang *et al*. 2009)	CCBAU 05354 (=KACC 14980=LMG 24661)^59^	105	[100]
*Parendozoicomonas verongulae* (Goldberg *et al*. 2018) Kim *et al*. 2024, 7^3^	comb. nov. (basonym: *Sansalvadorimonas verongulae* Goldberg *et al*. 2018)	RKSG058 (=ATCC TSD-72=LMG 29871)	147	[101]
*Paucisalibacillus algeriensis* Bendjama *et al*. 2014, 1362	sp. nov.	EB02 (=CSUR P858=DSM 27335)	34	[102]
*Pedobacter paludis* Zhang *et al*. 2019, 353^60^	sp. nov.	YX (=CCTCC AB 2018029=KCTC 62520)	62	[103]
*Peptoniphilus raoultii* Diop *et al*. 2019, 10^3^	sp. nov.	KHD4 (=CECT 9308=CSUR P0110)	72	[104]
*Phaeovulum molybdatiresistens* Song *et al*. 2026, 6^3^	sp. nov.	NW3 (=GDMCC 1.3054=JCM 35387)	142	[105]
*Photobacterium atrarenae* Kim *et al*. 2011, 436^61^	sp. nov.	M3-4 (=KCTC 23265=NCAIM B 02414)	126	[106]
*Planobacterium oryzisoli* Chhetri *et al*. 2023, 7^3,62^	sp. nov.	GCR5 (=KCTC 82713=TBRC 15746)^63^	27	[107]
*Polaribacter aestuariivivens* Park *et al*. 2019, 3^3^	sp. nov.	DBTF-3 (=KACC 19612=NBRC 113191)	66	[108]
*Pseudomonas hutmensis* Xiang *et al*. 2019, 876^64^	sp. nov.	XWS2 (=CCTCC AB 2018189=KACC 19898)	49	[109]
*Pseudomonas onubensis* Flores-Duarte *et al*. 2026, 10^3^	sp. nov.	L1=MIS C2 (=CECT 31157=LMG 34132)	106	[110]
*Pseudomonas pseudonitroreducens* Tohya *et al*. 2023, 1^3^	sp. nov.	BML-PP015 (=JCM 34578=LMG 32247)	124	[111]
*Pseudomonas qingdaonensis* Wang *et al*. 2019, 676^65^	sp. nov.	JJ3 (=JCM 32579=KCTC 62384)^66^	25	[112]
*Pseudomonas spartinae* Flores-Duarte *et al*. 2026, 10^3^	sp. nov.	SDT3 (=CECT 31003=LMG 34080)	106	[110]
*Pseudothermotoga profunda* (Mori *et al*. 2014) Belahbib *et al*. 2018, 561	comb. nov. (basonym: *Thermotoga profunda* Mori *et al*. 2014)	AZM34c06 (=DSM 23275=NBRC 106115)	53	[113]
*Pseudoxanthomonas fibrivorans* Yulei et al. 2026, 11^3,67^	sp. nov.	10 H (=CCTCC AB 2024091=KCTC 8654)	131	[114]
*Rhodococcus aromaticivorans* Muhammad *et al*. 2025, 14^3^	sp. nov.	DK17 (=InaCC B1662=KCCM 90599)	4	[115]
*Roseobacter pelophilus* Köpke 2007, 49^68^	sp. nov.	SAM4 (=DSM 17270=LMG 23018)	143	[116]
*Roseomonas aceris* Tonouchi and Tazawa 2014, 42	sp. nov.	R-1 (=DSM 26554=NBRC 109410)	38	[117]
*Rothia marina* Liu *et al*. 2013, 335	sp. nov.	JSM 078151 (=DSM 21080=KCTC 19432)	33	[118]
*Rubrivirga aquatilis* Han *et al*. 2025, 5^3,69^	sp. nov.	IMCC43871 (=KCTC 102072=NBRC 116463)^70^	140	[119]
*Rubrivirga halophila* Han *et al*. 2025, 6^3^	sp. nov.	IMCC45206 (=CCTCC AB 2023136=KCTC 92925=NBRC 116172)^71^	140	[119]
*Saliniferula* Yoon and Oh 2026, 5^3^	gen. nov.	*Saliniferula longa*	17	[120]
*Saliniferula longa* Yoon and Oh 2026, 6^3^	sp. nov.	KMU-117 (=KCCM 90342=NBRC 117094)	17	[120]
*Sediminibacterium soli* Wu *et al*. 2021, 972	sp. nov.	WSJ-3 (=CCTCC AB 2019408=KCTC 72839)	109	[121]
*Sinanaerobacter* Bao *et al*. 2021, 1614	gen. nov.	*Sinanaerobacter chloroacetimidivorans*	64	[122]
*Sinanaerobacter chloroacetimidivorans* Bao *et al*. 2021, 1614	sp. nov.	BAD-6 (=CCTCC AB 2021092=KCTC 25290)	64	[122]
*Snuella sedimenti* Kim *et al*. 2021, 5441^72^	sp. nov.	CAU 1569 (=KCTC 82409=MCCC 1K05670)	92	[123]
*Sphingobium salicis* Tournay *et al*. 2026, 15^3^	sp. nov.	WW5 (=DSM 120182=NCCB 101075)^73^	3	[124]
*Sphingomonas ginsenosidivorax* Jin *et al*. 2013, 1965^74^	sp. nov.	KHI67 (=JCM 17076=KACC 14951)^75^	35	[125]
*Spongitalea* Mitra *et al*. 2012, 383^76^	gen. nov.	*Spongitalea numazuensis*	117	[126]
*Spongitalea numazuensis* Mitra *et al*. 2012, 384	sp. nov.	HJ24 (=DSM 21243=NBRC 104581)	117	[126]
*Sporosarcina beigongshangi* Sun *et al*. 2022, 3^3^	sp. nov.	REN13 (=GDMCC 1.2151=JCM 34409)	86	[127]
*Stenotrophomonas oahuensis* Chuang *et al*. 2024, 8^3^	sp. nov.	A5586=D31 (=ICMP 25024=LMG 33201)	102	[128]
*Streptococcus bouchesdurhonensis* Zoaiter *et al*. 2023, 6^3^	sp. nov.	Marseille-Q6994 (=CSUR Q6994=DSM 113892)^24^	43	[129]
*Streptomyces composti* Duangupama *et al*. 2023, 9^3^	sp. nov.	SBST2-5 (=NBRC 113999=TBRC 9952)	63	[130]
*Streptomyces gilvigriseus* Ser *et al*. 2015, 1375	sp. nov.	MUSC 26 (=DSM 42173=NBRC 110931)^24,77^	100	[131]
*Streptomyces griseicoloratus* Xing *et al*. 2022, 5^3^	sp. nov.	TRM S81-3 (=CCTCC AA 2020002=LMG 31942)	121	[132]
*Streptomyces shinuiensis* Lim *et al*. 2025, 7^3^	sp. nov.	HK10 (=KACC 22137=NBRC 114904)	141	[133]
*Streptomyces tatrensis* Bielańska *et al*. 2026, 11^3^	sp. nov.	5.8 (=DSM 119354=PCM 3549)	148	[134]
*Sunxiuqinia dokdonensis* Chang *et al*. 2013, 744	sp. nov.	DH1 (=JCM 19380=KCTC 32503)^78^	40	[135]
*Sulfuriroseicoccus* Feng *et al*. 2022, 348^79^	gen. nov.	*Sulfuriroseicoccus oceanibius* ^80^	116	[136]
*Sulfuriroseicoccus oceanibius* Feng *et al*. 2022, 349^81^	sp. nov.	T37 (=KCTC 72799=MCCC 1H00391)	116	[136]
*Telluribacter roseus* Feng *et al*. 2023, 7^3^	sp. nov.	SYSU D00476 (=KCTC 82285=MCCC 1K04983)^82^	88	[137]
*Terrimonas suqianensis* Chen *et al*. 2017, 1065	sp. nov.	C3-5 (=CCTCC AB 2017042=KCTC 52676)	50	[138]
*Thermococcus radiotolerans* Jolivet *et al*. 2004, 225^83^	sp. nov.	EJ2 (=DSM 15228=JCM 11826)	41	[139]
*Thermodesulfobium fumaratoxidans* Maltseva *et al*. 2026, 11^3^	sp. nov.	4217–1 (=JCM 39391=KCTC 25626=VKM B-3675)	95	[140]
*Thiobacillus cuprinus* Huber and Stetter 1990, 321	sp. nov.	Hó5 (=DSM 5495=NBRC 102094)	110	[141]
*Thiobacillus prosperus* Huber and Stetter 1989, 484	sp. nov.	LM3 (=DSM 5130=JCM 30709)	137	[142]
*Thiobacter aerophilus* corrig. Dukat *et al*. 2024, 10^3,84^	sp. nov.	AK1 (=CGMCC 1.18099=UQM 41819)	96	[143]
*Thiobacteraceae* Dukat *et al*. 2024, 10^3^	fam. nov.	*Thiobacter*	96	[143]
*Tropicibacter alexandrii* Wang *et al*. 2020, 317	sp. nov.	LMIT003 (=CICC 24660=KCTC 62895)	91	[144]
*Uliginosibacterium sangjuense* Han *et al*. 2018, 263	sp. nov.	SS1-76 (=JCM 31375=KCTC 52159)	29	[145]
*Vibrio zhuhaiensis* Jin *et al*. 2013, 993^85^	sp. nov.	E20121 (=CCTCC AB 2011174=DSM 25602)	107	[146]
*Vitreoscilla massiliensis* Ndongo *et al*. 2021, 3317	sp. nov.	SN6 (=CSUR P2036=DSM 100958)^86^	56	[147]
*Xanthomonas hawaiiensis* Chuang *et al*. 2024, 6^3^	sp. nov.	A6251=D-93 (=ICMP 25022=LMG 33200)	102	[128]
*Xylophilus rhododendri* Lee *et al*. 2020, 4163	sp. nov.	CJ1-R5 (=CCTCC AB 2020030=KACC 21265)	19	[148]

For references to Validation Lists 1–71, see* Int J Syst Bacteriol ***49** (1999) 1325. Lists 72–197 were published in *Int J Syst Evol Microbiol ***50** (2000) 3, 423, 949, 1415, 1699, 1953; and **51** (2001) 1, 263, 793, 1229, 1619, 1945; and **52** (2002) 3, 685, 1075, 1437, 1915; and **53** (2003) 1, 373, 627, 935, 1219, 1701; and **54** (2004) 1, 307, 631, 1005, 1425, 1909; and **55** (2005) 1, 547, 983, 1395, 1743, 2235; and **56** (2006) 1, 499, 925, 1459, 2025, 2507; and **57** (2007) 1, 433, 893, 1371, 1933, 2449; and **58** (2008) 1, 529, 1057, 1511, 1993, 2471; and **59** (2009) 1, 451, 923, 1555, 2129, 2647; and **60** (2010) 1, 469, 1009, 1477, 1985, 2509; **61** (2011) 1, 475,1011, 1499, 2025, 2563; and **62** (2012) 1, 473, 1017, 1443, 2045, 2549; and **63** (2013) 1, 797, 1577, 2365, 3131, 3931; and **64** (2014) 1, 693, 1455, 2184, 3603; and **65** (2015), 1, 741, 1112, 2017, 2777, 3767; and **66** (2016) 1, 1603, 1913, 2463, 3761, 4299; and **67** (2017) 1, 529, 1095, 2075, 3140, 4291; and **68** (2018), 1, 693, 1411, 2130, 2707, 3379; and **69** (2019), 5, 597, 1247, 1844, 2627, 3313, and **70** (2020) 1, 1443, 2960, 4043, 4844, 5596. Lists 197–228 were published in* Int J Syst Evol Microbiol ***71** (2021) 004600, 004688, 004773, 004846, 004943, 005096; and **72** (2022) 005167, 005260, 005331, 005422, 005517, 005592; and **73** (2023) 005709, 005812, 005845, 005931, 005997, 006080; and **74** (2024) 006173, 006229, 006275, 006398, 006452, 006501; and **75** (2025) 006562, 006640, 006699, 006863, 006910, 006989; and **76** (2026) 007052.

1Abbreviations of culture collections cited in this list can be found at https://lpsn.dsmz.de/text/collections.

2Priority number assigned according to the date the documentation and request for validation are received.

3The journal in which the name was effectively published does not have continuous pages.

4The preferred etymology is Gr. masc. n. *zythos*, beer; N.L. gen. n. *zythi*, from beer.

5The etymology must state N.L. masc. adj. instead of N.L. neut. adj.

6The effective publication states that the type strain was also deposited as ACAM 1056, but no documentation was supplied.

7The etymology must state N.L. masc. adj.

8The list editors have corrected *alba* to *albus* (al’bus. L. masc. adj. albus, white).

9The list editors have corrected pacificus to pacificum (pa.ci’fi.cum. L. neut. adj. …).

10The effective publication states that the type strain was also deposited as KACC 23148, but no documentation was supplied.

11The preferred etymology is N.L. masc. adj. *bingmayongensis*, arbitrarily formed from the Chinese characters Bīng Mă Yŏng, ‘military servants’, ….

12Preferred syllabification and etymology are da.bao.shan.en’sis. N.L. masc. adj. *dabaoshanensis*, pertaining to the Dabaoshan Mine.

13The effective publication states that the type strain was also deposited as KCCM 90326, but no documentation was supplied.

14The preferred etymology is N.L. fem. n. *Bruguiera* ….

15Preferred syllabification and etymology are ther.mo.stel.la’tus. Gr. masc. adj. *thermos*, hot; L. masc. part. adj. stellatus, star-shaped; N.L. masc. part. adj. *thermostellatus*, referring to the formation of star-shaped aggregates at high temperatures.

16The effective publication states that the type strain was also deposited as DSM 18093, but no documentation was supplied.

17The list editors have corrected the epithet to *formosum* (for.mo’sum. L. neut. adj. *formosum*, …).

18The protologue erroneously has KCTC 42,606.

19The preferred etymology is N.L. gen. n. instead of M.L. gen. n.

20The type strain is mentioned in the abstract, but not in the protologue.

21The protologue heading must state *Clostridium* instead of C., and the etymology must state N.L. neut. adj. *tunisiense* instead of M.L. neut. adj. *tunisiense*.

22The CCBH number was erroneously given as 35,014. The effective publication states that the type strain was also deposited as CBAS 826, but no documentation was supplied.

23Preferred syllabification and etymology are bra.si.li.en’sis. N.L. masc. adj. *brasiliensis*, ….

24The authors gave DSMZ instead of DSM.

25The culture collection accession numbers are given in the abstract, but not in the protologue.

26The effective publication states that the type strain was also deposited as KACC 19451, but no documentation was supplied.

27Preferred syllabification and etymology are a.gri’co.la. L. masc. n. *agricola*, field dweller.

28The protologue heading must state *Frigoribacterium* instead of *F.*, and the syllabification must be corrected as follows: a.del’gis.

29The effective publication states that the type strain was also deposited as KCOM 1249, but no documentation was supplied.

30The preferred etymology is N.L. neut. adj. *periodonticum*, …, N.L. neut. adj. *pseudoperiodonticum*, …,

31The effective publication states that the type strain was also deposited as KCOM 1259, but no documentation was supplied.

32Preferred syllabification and etymology are bru.nes’cens. N.L. masc. part. adj. *brunescens*, becoming brown.

33Preferred syllabification and etymology are si.de.ro’phi.la. Gr. masc. n. *sideros*, iron; N.L. masc. adj. *philus*, loving; from Gr. adj. *philos*, loving; N.L. fem. adj. *siderophila*, iron-loving.

34The effective publication states that the type strain was also deposited as VKM A7094, but no documentation was supplied.

35The etymology must state L. fem. adj. instead of L. fam. adj.

36The preferred etymology is N.L. masc. adj.

37The etymology must state adj. instead of Adj.

38The preferred etymology is L. neut. n. *solum*, soil; L. fem. n. *silva*, forest.

39The protologue heading must state *Marinitalea* instead of *M*.

40The preferred etymology is L. fem. gen. pl. n. instead of L. fem. Gen. pl. n.

41Also misspelled AGBM02718 in the protologue.

42The preferred etymology is N.L. neut. n. *propanol*, propanol; ….

43The list editors have corrected the epithet *alcaliphilus* to *alkaliphilus* (al.ka.li’phi.lus. N.L. n. *alkali*, from Arabic *al-qaliy*, the soda ash; Gr. masc. adj. *philos*, loving; N.L. masc. adj. *alkaliphilus*, loving alkaline conditions).

44The etymology must state N.L. fem. adj. instead of N.L. masc. adj.

45In the abstract given as DSM 119677.

46In the protologue heading, the genus name is misspelled *Natrarchaebius*.

47In the abstract given as DSM 119936.

48The etymology must state gen. nov. instead of gen. Nov.

49The authors gave Luo *et al.* 2024 instead of Luo et al. 2025.

50The etymology must state L. fem. adj. *pacifica* instead of *pacificus*.

51The etymology must state N.L. masc. adj.

52The effective publication states that the type strain was also deposited as KCCM 101304, but no documentation was supplied.

53The list editors have corrected the epithet to jeddahensis (jed.dah.en’sis. N.L. masc. adj. *jeddahensis*, …).

54N’diop given in the protologue was also spelled Ndiop, NDiop and N’Diop in the article and in culture collection documents.

55Preferred syllabification and etymology are me.tal.li.re.du’cens. L. neut. n. *metallum*, …,

56The etymology must state N.L. masc. adj. instead of NL adj. masc.

57The list editors have corrected the epithet to *phocaeensis* (pho.cae.en’sis. N.L. masc. adj. *phocaeensis*, pertaining to Phocaea in Anatolia, home of the colonists that founded Massilia (Marseille).

58The etymology must state N.L. gen. n. instead of L. masc. adj.

59The effective publication states that the type strain was also deposited as HAMBI 3031, but no documentation was supplied.

60The etymology must state L. gen. n. *paludis*, of a swamp instead of L. gen. n. paludis (italic type) of a swamp.

61The protologue heading must state *Photobacterium atrarenae *instead of *P. atrarenae*.

62The etymology must state L. fem. n. *oryza*, rice; L. neut. n. *solum*, soil; N.L. gen. n. *oryzisoli*, of rice soil.

63The effective publication states that the type strain was also deposited as TISTR 2996, but no documentation was supplied.

64The list editors propose the following syllabification and etymology: hu.tmen’sis. N.L. fem. adj. *hutmensis*, arbitrarily formed from Hu-bei and Huang-jia-hu, where the strain was isolated and ‘tm’, abbreviation of traditional medicine.

65The etymology must state N.L. fem. adj. instead of N.L. msac. adj.

66The effective publication states that the type strain was also deposited as CGMCC 1.16493, but no documentation was supplied.

67The protologue heading must read *Pseudoxanthomonas fibrivoran*s sp. nov. instead of *Pseudxanthomonas fibrivorans* 10HT sp. nov.

68Preferred syllabification and etymology are pe.lo’phi.lus. Gr. masc. n. *pelos*, mud; Gr. masc. adj. *philos*, loving; N.L. masc. adj. *pelophilus*, mud-loving).

69The preferred etymology is L. fem. adj. *aquatilis*, aquatic, ….

70The effective publication states that the type strain was also deposited as HNIBRBA6996, but no documentation was supplied.

71The effective publication states that the type strain was also deposited as HNIBRBA14924, but no documentation was supplied.

72The etymology must state L. gen. n. *sedimenti* instead of L. gen. n. *sediment*.

73Culture collection numbers were not in the protologue but are found in the abstract and in the protologue of *Sphingobium salicis* subsp. salicis, for which validation was not requested.

74The preferred etymology is N.L. neut. n. *ginsenosidum*, … and N.L. fem. adj. *ginsenosidivorax*, ….

75The effective publication states that the type strain was also deposited as LMG 25801, but no documentation was supplied.

76The preferred etymology is L. fem. n. *spongia*, sponge.

77The effective publication states that the type strain was also deposited as MCCC 1K00504, but no documentation was supplied.

78The effective publication states that the type strain was also deposited as CGMCC 1.12676, but no documentation was supplied.

79The protologue heading must state *Sufuriroseicoccus* instead of sulfuriroseicoccus.

80The type species is not explicitly mentioned.

81The protologue heading must state *Sufuriroseicoccus oceanibius* instead of sulfuriroseicoccus oceanibius.

82The effective publication states that the type strain was also deposited as CGMCC 1.18647, but no documentation was supplied.

83The protologue heading must have *Thermococcus* instead of *T*. The preferred syllabification and etymology are ra.di.o.to’le.rans. L. masc. n. *radius*, a beam or ray; L. pref. *radio*-, pertaining to radiation; L. pres. part. *tolerans*, tolerating; N.L. masc. part. adj. *radiotolerans*, (γ-ray) radiation-tolerating.

84The list editors have corrected *aerophilum* to *aerophilus* (ae.ro’phi.lus. Gr. masc. n. aer, air; Gr. masc. adj. *philos*, loving; N.L. masc. adj. *aerophilus*, air loving).

85The preferred etymology is N.L. masc. adj. ….

86The effective publication states that the type strain was also deposited as LN870312, but no documentation was supplied.

## Corrigenda

The name *Nocardioides bruguierae* Chen *et al*. 2023 was listed both in Validation List no. 225 [149] and in Validation List no. 226 [150].

In Validation List no. 227 [151], the list editors corrected the name *Actinospongicola* Huang *et al*. 2025 to *Actinospongiicola*. In view of the latest emendation of Appendix 9, Section A (1)(c) of the ICNP, this correction was not necessary.

## Abbreviations

ICNP: International Code of Nomenclature of Prokaryotes
IJSEM: International Journal of Systematic and Evolutionary Microbiology
